# Innovative Biochar-Coated Cocoon-Based Adsorbent for the Removal of Organic and Inorganic Pollutants from Water

**DOI:** 10.3390/ma19214512

**Published:** 2026-10-27

**Authors:** Anna Marszałek, Aneta Magdziarz, Ewa Puszczało, Mariusz Dudziak, Gabriela Kamińska

**Affiliations:** 1 Department of Water and Wastewater Engineering, Silesian University of Technology, Stanisława Konarskiego St. 18, 44-100 Gliwice, Poland; anna.marszalek@polsl.pl (A.M.); mariusz.dudziak@polsl.pl (M.D.); 2 Department of Heat Engineering & Environment Protection, Faculty of Metals Engineering and Industrial Computer Science, AGH University of Krakow, Al. Mickiewicza 30, 30-059 Krakow, Poland

**Keywords:** biochar, biomass-derived adsorbent, silkworm cocoon, adsorption, Pb(II) removal, phenol removal, water treatment

## Abstract

The present study describes the development of an innovative adsorbent based on Bombyx mori silkworm cocoons for the removal of organic and inorganic pollutants from water. The material was prepared by coating raw silkworm cocoons with biochar derived from the same biomass through pyrolysis under nitrogen followed by KOH activation and subsequently incorporating the coated cocoons into a sodium alginate matrix using dip-coating and calcium crosslinking. The materials were characterised using SEM, BET, DFT, FTIR, and TGA analyses. KOH activation substantially improved the porosity of biochar, increasing its specific surface area from 1.56 to 1178 m^2^/g and producing a predominantly microporous structure. The final biochar-coated cocoon compound exhibited a specific surface area of 422.29 m^2^/g. SEM observations confirmed the preservation of the fibrous cocoon morphology after coating, while FTIR and TGA analyses provided information on surface functional groups and thermal behaviour. Adsorption performance was evaluated for Pb(II) and phenol under static and dynamic conditions. The Langmuir model provided a slightly better fit for Pb(II) adsorption than the Freundlich model, whereas phenol adsorption was more clearly described by the Langmuir model. Dynamic column experiments showed higher removal efficiency and longer breakthrough times for Pb(II) than for phenol. A single regeneration cycle resulted in partial recovery of adsorption performance, particularly for Pb(II), although further consecutive adsorption–desorption cycles are needed to establish long-term reusability and stability. Overall, the results demonstrate the potential of this cocoon-based biochar composite as a sustainable adsorbent for water treatment, particularly for Pb(II) removal under batch and flow conditions.

## 1. Introduction

Water pollution with heavy metals and organic compounds such as phenol is a major environmental problem of global concern. Lead ions are among the most toxic heavy metals, and their presence in drinking water can lead to neurological, renal, and developmental disorders in humans [1]. Phenol, on the other hand, is a toxic organic pollutant that is difficult to biodegrade and may exert mutagenic and carcinogenic effects [2]. For this reason, the effective removal of these pollutants from water and wastewater remains one of the key challenges in environmental protection. Among the various water treatment methods, including chemical precipitation, ion exchange, membrane filtration, and electrochemical processes, adsorption is widely recognized as an effective approach for the removal of a broad range of pollutants [3]. Adsorption can also be integrated with other treatment technologies, such as membrane filtration, to enhance overall treatment performance [4]. In particular, adsorption-based approaches are widely investigated for the removal of heavy metals from wastewater, including lead and other potentially toxic metal ions [5]. The possibility of adsorbent regeneration and reuse further enhances the practical potential of adsorption-based treatment processes [6]. The performance of adsorption is strongly influenced by the physicochemical properties of the adsorbent, particularly its specific surface area, pore structure, and the presence of surface functional groups capable of interacting with pollutant molecules [7]. In recent years, there has been a growing interest in biochars, which are a cheap, renewable and environmentally friendly adsorption materials obtained in the process of biomass pyrolysis [8,9]. Their advantages are high chemical stability, the possibility of structural modification and a high pore content. In order to improve their sorption properties, chemical activation is often used, especially with the use of potassium hydroxide (KOH), which increases the specific surface area, develops microporosity and introduces oxygen groups conducive to adsorption [10,11]. Parameters of the activation process, such as temperature and the ratio of KOH to biomass, have a key impact on the development of the porous structure and the nature of the surface [12].

So far, most studies on biochars have focused on plant-based materials (e.g., rice husks, coconut shells, sawdust), while increasing attention has been given to protein-rich biomass as an alternative carbon precursor [13,14,15]. Protein-based materials, such as feathers, chitosan and silk fibroin, have been investigated for the production of carbonaceous adsorbents with diverse surface chemistry and adsorption properties [16]. Their composition and structure may contribute to the development of surface functionalities during carbonization and activation, which can influence the interaction of carbon materials with both metal ions and organic compounds. In this context, silkworm cocoons, which are waste from the textile industry, are an interesting source of biomass with a high protein content (fibroins and sericins). Their conversion into activated carbon material can be an example of the efficient use of organic waste in the spirit of the circular economy [17,18]. Despite this, the number of reports of KOH-activated biochars obtained from silkworm cocoons is still limited. Few studies indicate their effectiveness in removing dyes or metal ions, and fewer concern their composites with natural polymers, such as sodium alginate [19].

This paper presents an innovative approach to the dual use of animal biomass waste *Bombyx mori* silkworm cocoons as both: (1) a precursor for the synthesis of high-efficiency, microporous biochar with a large specific surface area and as (2) a support material coated with the obtained biochar via dip-coating in a sodium alginate matrix. Sodium alginate, a natural polysaccharide extracted from brown algae, has numerous carboxyl and hydroxyl groups that can bind heavy metals and organic compounds [20]. In the presence of calcium ions, it forms stable hydrogels, which allows the immobilization of activated carbon particles and obtaining composites with good mechanical stability and reusability [21,22]. The combination of biochar with an alginate matrix not only increases the adsorption efficiency but also improves the practical properties of the material in columnar processes as the final adsorbent has the mechanically stable and regenerated form. To the best of our knowledge, this concept is proposed here for the first time, as existing studies have mainly focused on powdered biochar derived from silkworm cocoons. Comprehensive material characterization, including surface analysis (BET), functional group identification (FTIR) and thermal stability assessment (TG/DTG), allowed to visualize the relationship between activation conditions and porous structure and surface functionality.

Adsorption studies were conducted under both static and dynamic conditions, which is a significant extension compared to the previous literature reports. For batch adsorption experiments, isothermal (Langmuir, Freundlich) and kinetic (pseudo-second-order) models were used, allowing for a detailed analysis of the sorption mechanism. In the dynamic adsorption studies, Thomas models were used, which allowed for a quantitative assessment of the course of the breakthrough curve, adsorption rate and column capacitance.

In addition, the adsorbent’s regeneration was evaluated, confirming its high stability and reusability without significant loss of efficiency. The results confirm that the proposed biocomposite represents a new generation of sustainable adsorbents, combining biological origin, high surface activity and mechanical resistance, which opens up the prospects for its use in water treatment systems on a real scale.

## 2. Materials and Methods

### 2.1. Fabrication of Biochar-Coated Cocoon-Based Adsorbent

#### 2.1.1. Biochar Production

Silkworm cocoons were cut into small pieces and directly carbonized in a muffle furnace at 500 °C for 30 min. The resulting product was mixed with potassium hydroxide (KOH, 1 M) at a mass ratio of 1:1 and subsequently activated in a tubular furnace at 800 °C under a nitrogen atmosphere. The activation process was carried out for 2 h with a heating rate of 10 °C min^−1^. After cooling to room temperature, the product was washed with 4% aqueous hydrochloric acid (HCl) and repeatedly rinsed with deionized water until a neutral pH was achieved. Finally, it was dried at 80 °C.

#### 2.1.2. Production of Biochar-Coated Cocoon-Based Adsorbent

**Figure 1:**
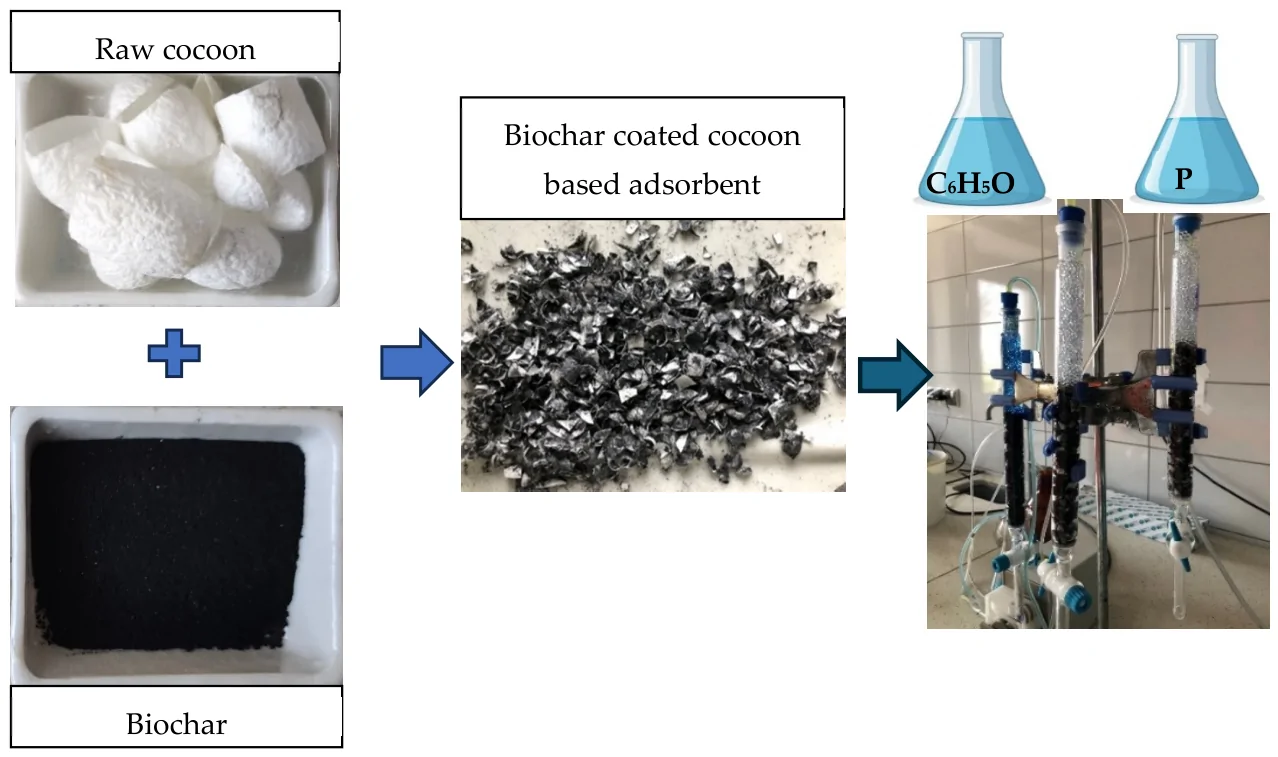
Schematic illustration of the synthesis of biochar-coated cocoon-based adsorbent and its subsequent application for the removal of Pb(II) ions and phenol from aqueous solutions.

First, 2 g of sodium alginate was dissolved in 100 mL of deionized water and stirred for 24 h. Then, 2.5 g of the obtained biochar was added to 70 mL of the sodium alginate solution and homogenized using an ultrasonic disintegrator for 10 min in pulsed mode (10 s ON/5 s OFF). Raw silkworm cocoons (7 g) were immersed in the prepared suspension for 2 h to allow the biochar coating to adhere to the cocoon surface. The coating was performed using the dip-coating method. The coated cocoons were dried at 40 °C for 24 h and subsequently immersed in a 1% CaCl_2_ solution for 10 min to crosslink the sodium alginate matrix. Finally, the composite was rinsed with deionized water, dried again at 40 °C, and cut into approximately 5 mm pieces (Figure 1).

#### 2.1.3. Chemical Analysis

All materials used were purchased as analytical-grade reagents and used without further purification. Sodium alginate, EDTA, HNO_3_, NaOH, CaCl_2_ and phenol were purchased from Sigma-Aldrich (Poznań, Poland). Lead ion and phenol concentrations were monitored spectrophotometrically with Merck test kits. Feed water solution was prepared based on Milli-Q water spiked with lead and phenol as adsorbates. Lead standard solutions (PbNO_3_) were purchased from Merck (Merck sp. z o.o., Warsaw, Poland).

### 2.2. Methods of Characterization of Biochar

The morphology of the samples was examined using scanning electron microscopy (SEM). The images were obtained at various magnifications to analyze the surface structure and changes after carbonization and activation processes.

The textural characterization of the tested materials (raw silk cocoon, carbonized cocoon, biochar, biochar coated cocoon-based adsorbent) was carried out on the basis of nitrogen adsorption–desorption isotherms at 77 K, using the Anton Paar Nova 800 specific surface area analyzer. Prior to analysis, the samples were degassed at 60 °C for 12 h under vacuum conditions to remove adsorbed impurities and moisture. The specific surface area was determined by the Brunauer–Emmett–Teller method (BET) on the basis of the adsorption branch of the isotherm in the range of relative pressures p/p_0_ = 0.05–0.30. The total pore volume was determined by the amount of adsorbed nitrogen at p/p_0_ ≈ 0.99. The pore size distribution and volume were determined using the DFT (Non-Local Density Functional Theory) method, using a model of nitrogen adsorption on carbon materials (cylindrical pores).

The surface functional groups of the studied samples (raw silk cocoon, carbonized cocoon, biochar) were analysed using Fourier-transform infrared spectroscopy (FTIR). The measurements were performed using a Bruker ALPHA FTIR spectrometer in the wavenumber range of 4000–400 cm^−1^, with a spectral resolution of 4 cm^−1^ and 32 scans collected for each sample. The spectra were recorded in absorbance mode.

The skeletal density of the obtained materials was determined using an Ultrapyc 5000 Micro gas pycnometer (Anton Paar, Graz, Austria). The measurements were performed using the helium displacement method. Prior to analysis, the samples were dried to remove moisture, placed in the measurement chamber, and purged with helium. The density of each material was calculated from the sample mass and the skeletal volume determined by helium displacement.

The thermal stability of the biochars was evaluated using thermogravimetric analysis (TGA) with a Mettler Toledo (Greifensee, Switzerland) TGA/DSC Star System analyzer. The analyses were conducted in an air atmosphere (40 mL/min) by heating the samples from room temperature to 950 °C at a constant heating rate of 10 °C/min. Prior to analysis, the samples were ground to a particle size below 500 µm. Each measurement was performed in triplicate. The thermal behaviour of the samples was analyzed based on TG and derivative thermogravimetric (DTG) curves, and the temperature of maximum mass loss (T_p_) was determined.

The zeta potential of the biochar-coated cocoon-based adsorbent was determined using a SurPASS 3 electrokinetic analyzer (Anton Paar, Graz, Austria) based on the streaming potential method. The measurements were performed at room temperature using an adjustable-gap measuring cell. The zeta potential was calculated from the streaming potential according to the Helmholtz-Smoluchowski equation. Each sample was measured in triplicate, and the results are reported as the mean ± standard deviation. The zero point charge measurements of (pH pzc) of adsorbent were performed as follows: pH (from 2 to 12) was adjusted using 0.1 M HNO_3_/NaOH solutions, monitored by a multifunctional analyzer CPC-505 (Elmetron, Zabrze, Poland). For this purpose, 50 mg of the adsorbent was added to 50 mL of distilled water at pHs from 2 to 11, stirred at room temperature for 24 h, and the solution pH levels measured. The difference between the initial and final pH values (ΔpH = pHf − pHi) for each series was plotted as a function of pHi. Then the pH of PZC was estimated at the point where it crossed ΔpH (pHi − pHf) = 0.0 [23].

### 2.3. Static Adsorption

Static adsorption experiments were conducted at 20 °C in 100 mL glass flasks shaken at 300 rpm. Adsorption isotherms were determined using initial Pb(II) concentrations of 2, 5, 10, 20, and 60 mg/L and initial phenol concentrations of 2, 5, 10, 20, and 50 mg/L. The adsorbent dosages were 5 g/L for Pb(II) and 15 g/L for phenol. The contact time was 60 min. The effect of solution pH on adsorption was investigated separately using a solution volume of 50 mL, with the pH adjusted using 0.1 M HNO_3_ and NaOH solutions.

Kinetic experiments were conducted at 20 °C and a shaking speed of 300 rpm. The initial Pb(II) and phenol concentrations were 1.26 and 1.5 mg/L, respectively. The adsorbent dosages were 5 g/L for Pb(II) and 14 g/L for phenol. Samples were collected after 10, 20, 30, 60, 120, 180, and 240 min to determine the residual pollutant concentrations.

### 2.4. Adsorption Isotherms and Kinetic Model

The adsorption equilibrium data were analyzed using the Langmuir and Freundlich isotherm models, while the adsorption kinetics were evaluated using the pseudo-second-order kinetic model. A detailed description of the mathematical equations, model assumptions, and fitting procedure has been provided in our previous work [24] and is therefore not repeated here.

### 2.5. Dynamic Adsorption

Dynamic studies were carried out using adsorption columns with a height of 20 cm (bed height equal to 14 cm) and a diameter of 1.5 cm. Two columns were connected separately to feed water containing lead with concentration of 1 mg/L and the other to phenol (1 mg/L). The feed water was transported to the columns by a peristaltic pump. The flow rate was 2 mL/min. The adsorption columns were regenerated for 1 h using 0.01 M EDTA for Pb(II) and 0.1 M NaOH for phenol. The results obtained in column experiments were used to construct the breakthrough curves by plotting C_t_/C_0_ against time_._ The breakthrough point was defined as the moment when the effluent concentration reached a 10% and non-conventionally 50% (due to high values of phenol concentration at initial stage of column operation) of the influent concentration. The exhaustion time was defined as the moment when the effluent concentration reached a 90% of the influent concentration. Experimental data were described using Thomas model according to Equation (1):

(1) $$\frac{C_{t}}{C_{0}}=\frac{1}{1+\exp\left(\frac{k_{\mathrm{Th}}}{Q}\cdot (q_{\mathrm{Th}}\cdot m-C_{0}\cdot Q\cdot t\right)}$$

where *k_Th_* is the Thomas rate constant (L·h^−1^·mg^−1^), *q_Th_* is the equilibrium adsorbate uptake per g of the adsorbent (mg·g^−1^), *m* is the mass of the adsorbent in the column (g), *C*_0_ is the initial adsorbate concentration (mg·L^−1^), *C_t_* is the effluent concentration at time *t* (mg·L^−1^), *Q* is the flow rate (L·h^−1^), *t* is the effluent collection time (h) [25].

### 2.6. Statistical Analysis

All experiments were performed in triplicate, and the results are presented as the mean standard deviation. The experimental data were analyzed using Microsoft Excel 2021 (Microsoft Corp., Redmond, WA, USA). The goodness of fit of the adsorption isotherm, kinetic, and breakthrough curve models was evaluated using the coefficient of determination R^2^.

## 3. Discussion of Research Results

### 3.1. Adsorbent Characteristics

The obtained materials were comprehensively characterized to evaluate their physicochemical properties. SEM analysis was used to investigate morphology, while BET and DFT methods were applied to determine textural parameters. FTIR spectroscopy provided information on surface functional groups, and TGA analysis was used to assess thermal stability. Additionally, true density was determined using helium pycnometry. These results were used to interpret the adsorption performance of the materials.

**Figure 2:**
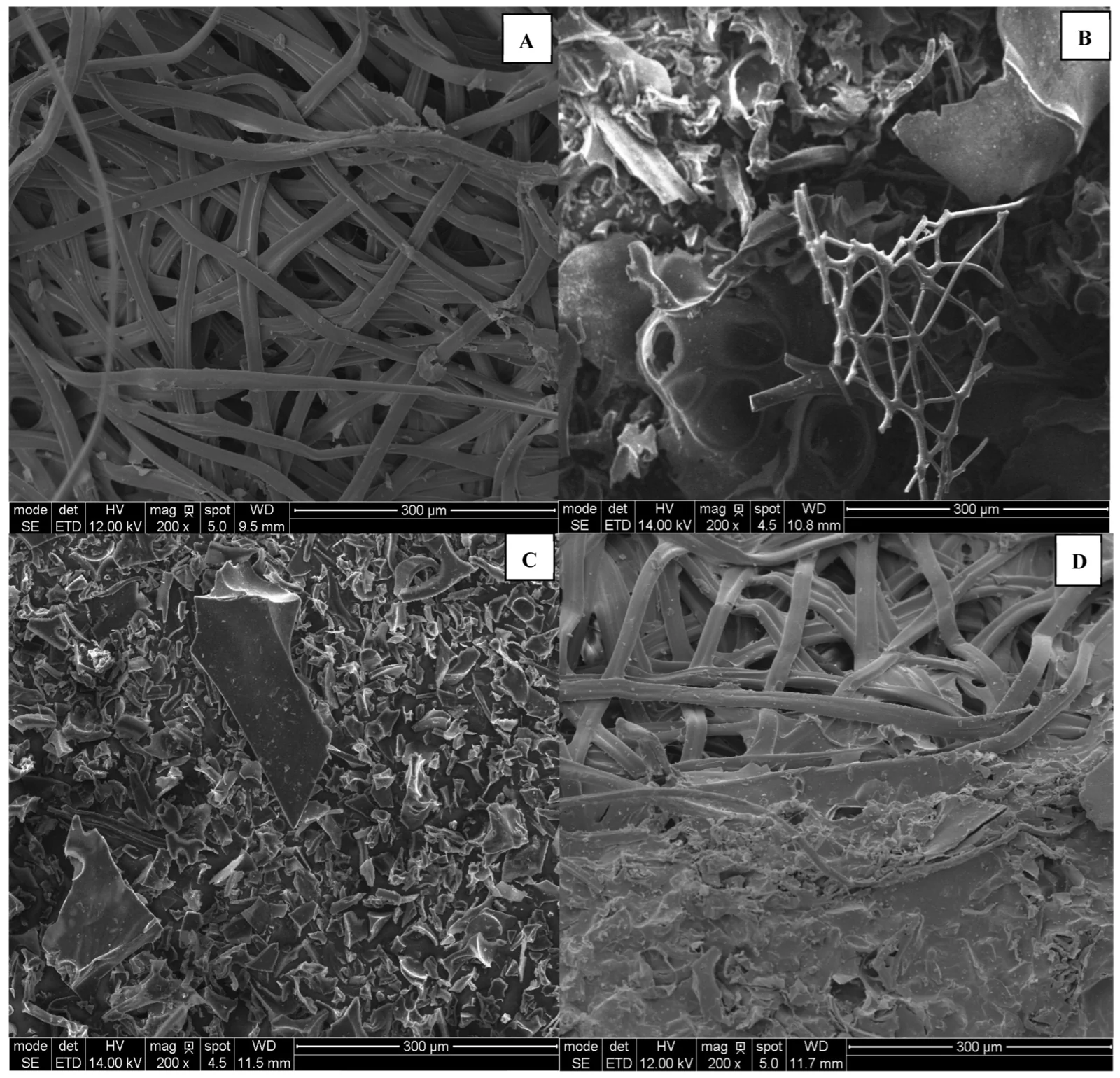
SEM images of silkworm cocoon-derived materials: (**A**) raw silk cocoon, (**B**) carbonized cocoon, (**C**) biochar, and (**D**) biochar coated cocoon-based adsorbent.

SEM images show significant morphological changes occurring during successive processing stages (Figure 2).

SEM images show significant morphological changes occurring during successive processing stages. The raw silk cocoon exhibits a well-defined fibrous structure with a smooth surface.

After carbonization, the fibrous morphology is partially preserved, although the surface becomes more compact and rougher, indicating the formation of a carbonaceous structure. The biochar sample shows a highly developed and irregular surface with numerous defects and roughness, resulting from chemical etching and pore formation. In contrast, the carbon-coated cocoon retains its fibrous structure, but the surface is covered with a carbon layer, leading to increased surface roughness. These observations are consistent with BET analysis, confirming the significant development of surface area after chemical activation.

The specific density of the tested materials varied depending on the method of obtaining them. For biochar obtained at 500 °C, an average density of 1.6810 g/cm^3^ was obtained. For the chemically activated material (KOH, 800 °C), this value increased to 1.9382 g/cm^3^, while for the carbon-coated cocoon it was 1.6568 g/cm^3^. The results are presented in Table 1. The observed increase in the specific density of the activated material KOH at 800 °C may be due to the intensification of carbonization processes and reorganization of the carbon structure, leading to the formation of a more ordered and condensed carbon matrix. At the same time, chemical activation promotes the development of a porous structure, but the measurement of specific density (helium) refers to the skeletal volume of the material, which does not take into account the volume of open pores, which explains the observed increase in density values. Lower density values for 500 °C carbonized biochar and biochar-coated cocoon may indicate a less developed carbon structure and a higher proportion of amorphous phases or organic residues.

The obtained results demonstrate the significant influence of both carbonization and chemical activation on the development of the porous structure (Table 2). The carbonized silkworm cocoon exhibited a very low specific surface area (1.560 m^2^/g) and negligible pore volume (0.0026 cm^3^/g), indicating a poorly developed porous structure with predominantly mesopores. In contrast, KOH activation at 800 °C dramatically increased the specific surface area to 1178 m^2^/g and the total pore volume to 0.539 cm^3^/g, resulting in a predominantly microporous material with an average pore diameter of 1.93 nm. The nitrogen adsorption—desorption isotherm of the activated biochar corresponded to a Type I isotherm, confirming the dominance of micropores. Following dip-coating of the activated biochar onto the silkworm cocoon using sodium alginate as a binder, the specific surface area decreased to 422.29 m^2^/g, while the pore volume decreased to 0.1723 cm^3^/g and the average pore diameter to 1.43 nm. This corresponds to a 64.2% decrease in specific surface area, which may be associated with partial micropore blockage and reduced pore accessibility following alginate-based immobilization. This decrease may be associated with partial pore blocking or reduced pore accessibility resulting from alginate-based immobilization of the biochar on the cocoon fibers. However, the available data do not allow us to determine whether micropores are preferentially blocked. Nevertheless, the composite retained a well-developed microporous structure suitable for adsorption applications. The nitrogen adsorption-desorption isotherm of the final biochar-coated cocoon has been provided as Supplementary Figure S1.

**Figure 3:**
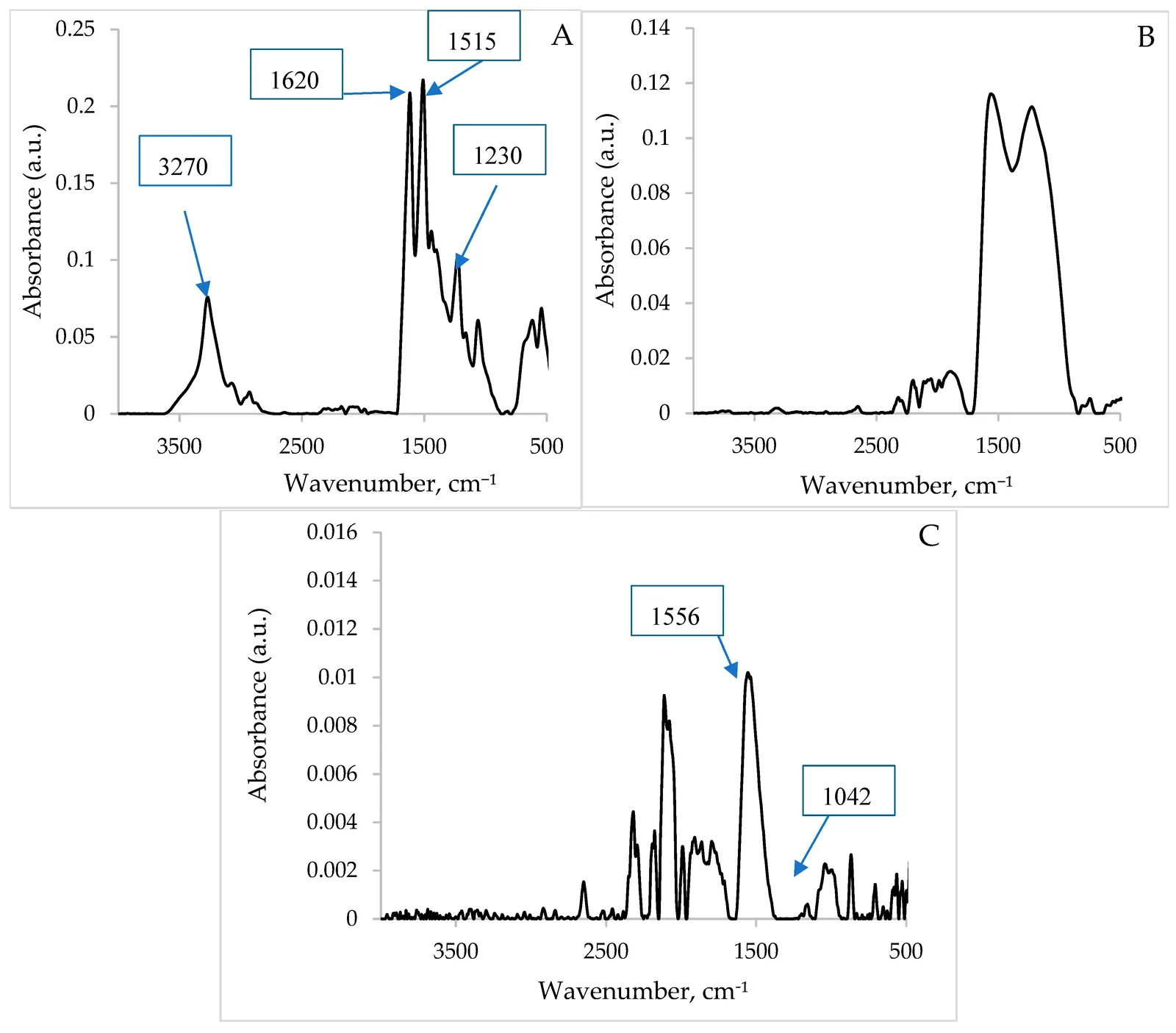
FTIR spectra of silkworm cocoon-derived materials: (**A**) raw silk cocoon, (**B**) carbonized cocoon, and (**C**) biochar.

The FTIR spectra of the silkworm cocoon-derived materials, including the raw silkworm cocoon, carbonized cocoon (500 °C), and KOH-activated biochar (800 °C), are presented in Figure 3.

The spectrum of the raw silkworm cocoon exhibited a broad absorption band at approximately 3270 cm^−1^, assigned to the stretching vibrations of hydroxyl (O-H) and amine (N-H) groups. Characteristic absorption bands located at approximately 1620, 1515, and 1230 cm^−1^ correspond to amide I, amide II, and amide III vibrations, respectively, confirming the proteinaceous structure of fibroin and sericin [26,27]. Following carbonization at 500 °C, the characteristic amide bands markedly decreased in intensity, indicating the thermal decomposition of fibroin and sericin and the gradual formation of a carbon-rich structure. Simultaneously, the broad O-H/N-H absorption band became less intense, reflecting the removal of volatile components and the structural changes occurring during carbonization [28].

The FTIR spectrum of the KOH-activated biochar differed markedly from those of the raw and carbonized cocoon. The characteristic protein-related bands almost completely disappeared, confirming the extensive transformation of the silk precursor during carbonization and subsequent chemical activation. The dominant absorption band at 1556 cm^−1^ was assigned to the stretching vibration of aromatic C=C bonds, indicating the formation of condensed aromatic carbon structures during carbonization and KOH activation. Weak absorption bands at 1159 and 1042 cm^−1^ were attributed to C-O stretching vibrations of residual oxygen-containing functional groups [29]. Overall, the FTIR spectra confirmed substantial chemical changes occurring during the successive stages of material preparation. The progressive disappearance of the characteristic protein bands, together with the appearance of bands associated with aromatic carbon structures, demonstrates the successful conversion of the silkworm cocoon into activated biochar. These observations are consistent with the BET analysis, which revealed a significant increase in the specific surface area and the development of a predominantly microporous structure after KOH activation. The formation of an aromatic carbon framework together with the presence of residual oxygen-containing functional groups may contribute to the adsorption performance of the obtained biochar.

**Figure 4:**
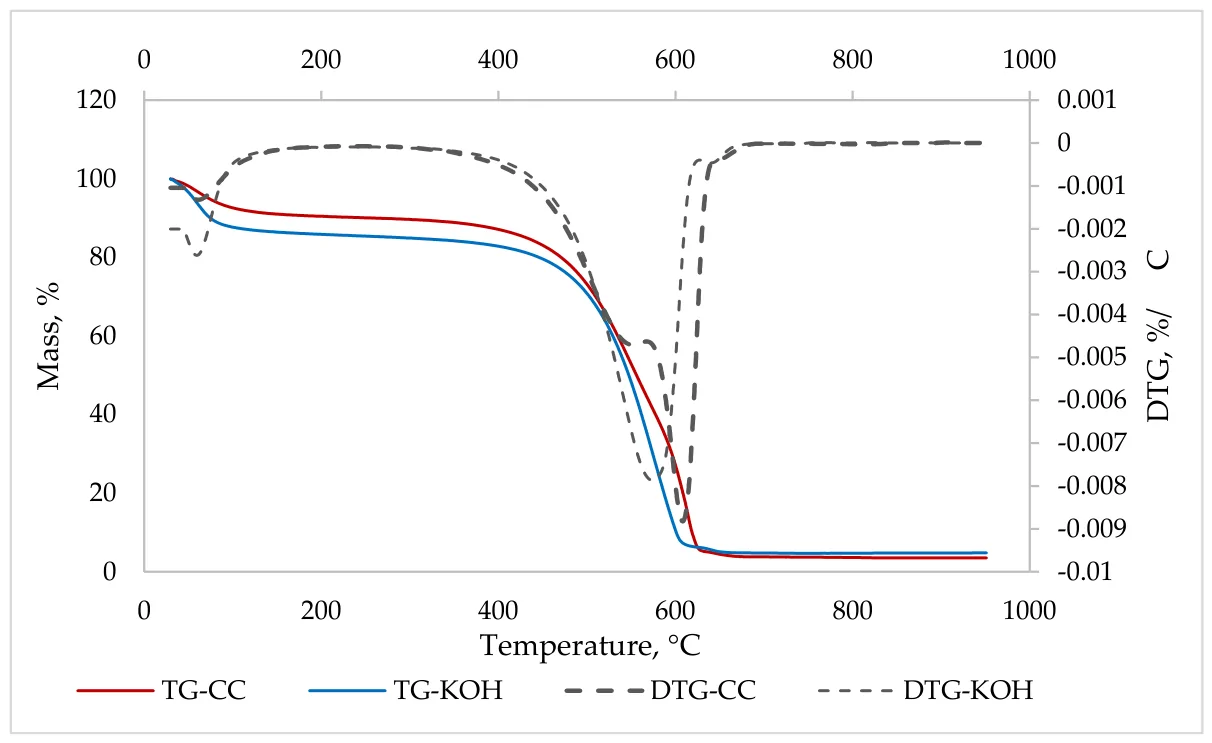
TG and DTG curves of biochar obtained at carbonised cocoon (TG-CC, DTG-CC) and biochar (TG-KOH, DTG-KOH).

Thermogravimetric analysis revealed clear differences in the thermal behaviour of the materials (Figure 4). The biochar obtained at 500 °C exhibited a broader DTG peak at approximately 600–620 °C, indicating a more heterogeneous and less ordered structure. In contrast, the activated biochar showed a sharper DTG peak shifted to lower temperatures (570–590 °C), suggesting increased reactivity associated with a highly developed porous structure. This shift can be explained by enhanced oxygen accessibility within the porous network. Both samples exhibited similar residual mass (5%), indicating comparable mineral content.

The TG and DTG curves (Figure 4) show that the main thermal decomposition for both materials occurs in the range of 500–650 °C. The higher reactivity of the activated biochar is consistent with its significantly larger surface area and microporous structure, which facilitate oxidation processes. Similar behaviour has been reported for chemically activated biochars, where increased porosity and structural defects lead to enhanced reactivity [30].

These structural and thermal characteristics are directly reflected in adsorption performance. The developed porosity of the activated biochar provides a greater number of active sites, resulting in improved adsorption capacity, particularly for Pb(II), where electrostatic interactions and surface complexation play a dominant role [31].

### 3.2. Effect of pH on Adsorption and Zeta Potential of the Adsorbent

**Figure 5:**
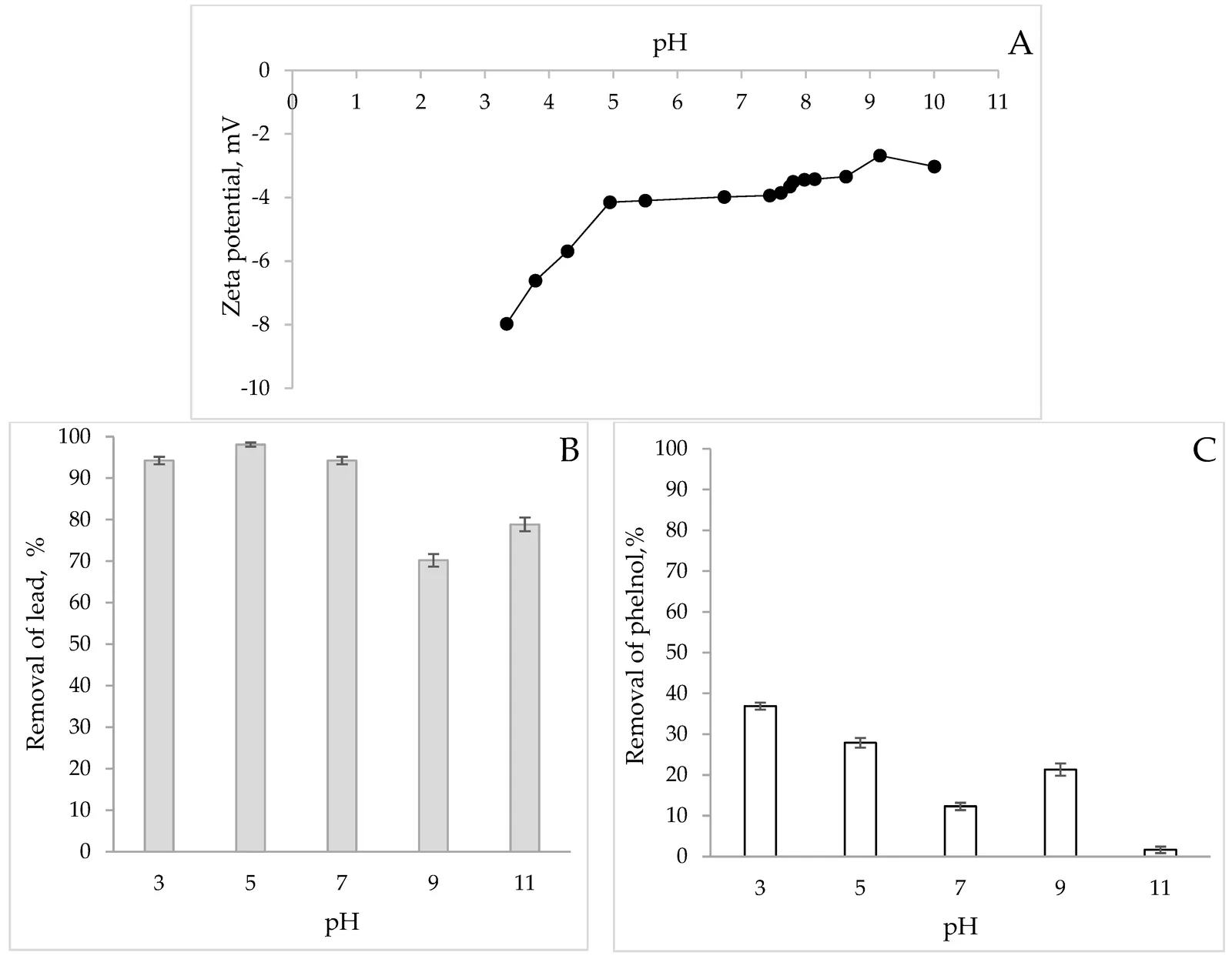
Zeta potential of the biochar-coated cocoon-based adsorbent (**A**) and effect of initial solution pH on the removal efficiency of Pb(II) (**B**) and phenol (**C**). Adsorption experiments were conducted at room temperature for 60 min under stirring at 300 rpm, using 50 mL of solution. The initial pollutant concentrations and adsorbent dosages are specified in the experimental conditions.

The effect of feed water solution pH on the adsorption of Pb(II) and phenol was investigated to evaluate the role of surface charge in the adsorption process (Figure 5). The pH of the feed water influences both the speciation of the adsorbates and the surface properties of the adsorbent. Therefore, zeta potential and the point of zero charge measurements were performed to determine the surface charge of material and to better understand the electrostatic interactions governing adsorption (Figure 5). The zeta potential curve revealed that, under acidic conditions, the adsorbent surface exhibited a negative charge, which gradually increased with increasing pH, reaching a weak negative value of −2.67 mV at pH 9.15. This result is in line with the point of zero charge (pHpzc) of the sorbent, determined as pHpzc = 9.8 using the acid-base potentiometric titration method.

Although the adsorbent surface was negatively charged throughout the investigated pH range, the adsorption of Pb(II) decreased under alkaline conditions. This behavior cannot be explained by surface charge alone and may be influenced by changes in Pb(II) speciation, hydrolysis, and the possible formation of lead hydroxide precipitates. Therefore, electrostatic attraction may contribute to Pb(II) uptake, but its effect should be considered together with other pH dependent processes. The contribution of Pb(OH)_2_ precipitation was not independently verified.

On the other hand, phenol adsorption decreased gradually with increasing pH. Under acidic conditions, phenol predominantly exists in its neutral molecular form, which may facilitate π–π interactions with aromatic domains of the biochar and hydrogen bonding with surface functional groups. As the pH increases, partial deprotonation of phenol to phenolate ions (C_6_H_5_O^−^) may contribute to the observed decrease in adsorption due to electrostatic repulsion from the negatively charged adsorbent surface. These results indicate that acidic conditions favor the adsorption of both Pb(II) and phenol, although the underlying mechanisms may differ. Surface complexation and electrostatic attraction may contribute to Pb(II) adsorption, whereas π–π interactions and hydrogen bonding may contribute to phenol adsorption. These mechanisms are proposed based on the observed adsorption behavior and the physicochemical characteristics of the adsorbent; however, they were not directly confirmed by post-adsorption spectroscopic analysis.

### 3.3. Adsorption Isotherms and Adsorption Kinetics of Pb(II) and Phenol

To better understand the adsorption behavior of Pb(II) and phenol, the adsorption kinetics were investigated by monitoring the adsorption capacity (Qt) as a function of contact time. The kinetic data were further analyzed using the pseudo-second-order model.

**Figure 6:**
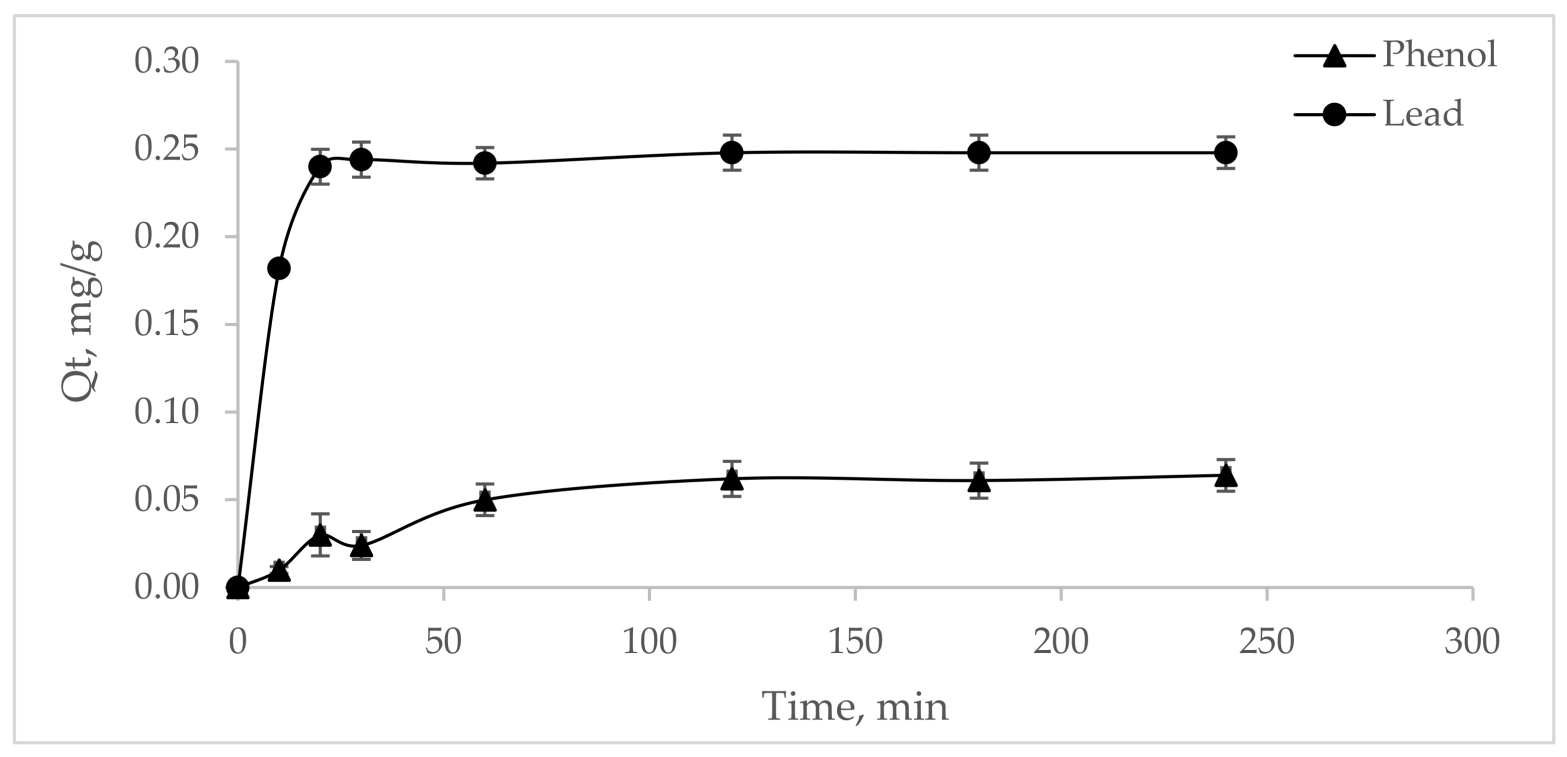
Adsorption kinetics of phenol and lead on the biochar-coated cocoon-based adsorbent. Experimental conditions: temperature 20 °C; initial Pb(II) concentration 1.26 mg/L; initial phenol concentration 1.5 mg/L; adsorbent dosage 5 g/L for Pb(II) and 14 g/L for phenol; shaking speed = 300 rpm. Error bars represent standard deviations of triplicate measurements.

As shown in Figure 6, the adsorption kinetics differed considerably between Pb(II) and phenol. Pb(II) adsorption proceeded rapidly during the initial stage, reaching approximately 0.24 mg/g within 20 min, followed by a nearly constant adsorption capacity. At 60 min, the adsorption capacity was approximately 0.24 mg/g, compared with 0.25 mg/g at longer contact times. In contrast, phenol adsorption proceeded more gradually, increasing from approximately 0.01 mg/g at 10 min to 0.05 mg/g at 60 min and approximately 0.06 mg/g at 240 min. These results indicate that the selected contact time of 60 min was sufficient for Pb(II) adsorption to approach a plateau, whereas phenol adsorption continued gradually beyond this period. Therefore, the 60 min contact time used in the subsequent batch experiments represents an operational condition rather than an equilibrium time for both adsorbates.

**Figure 7:**
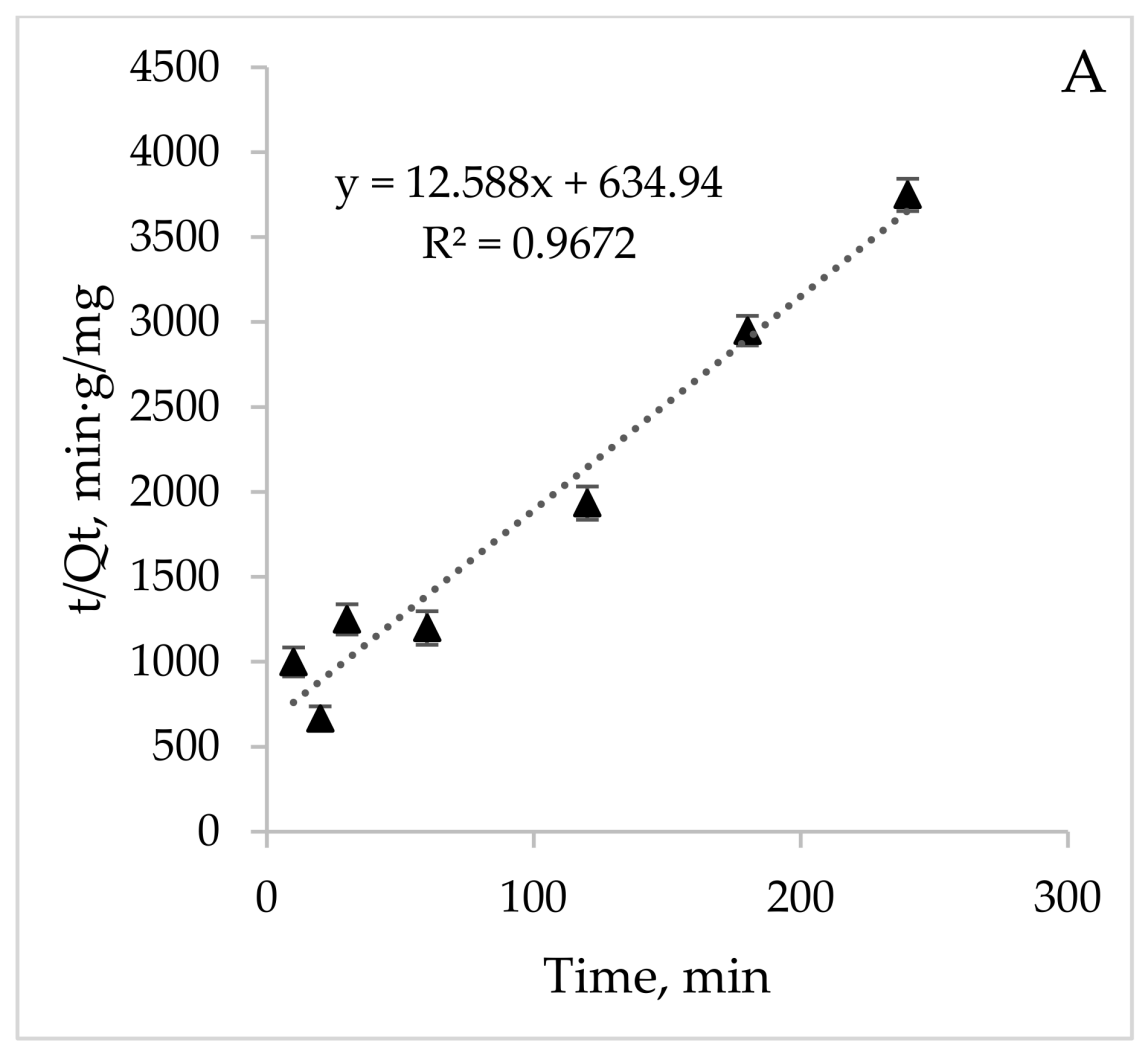

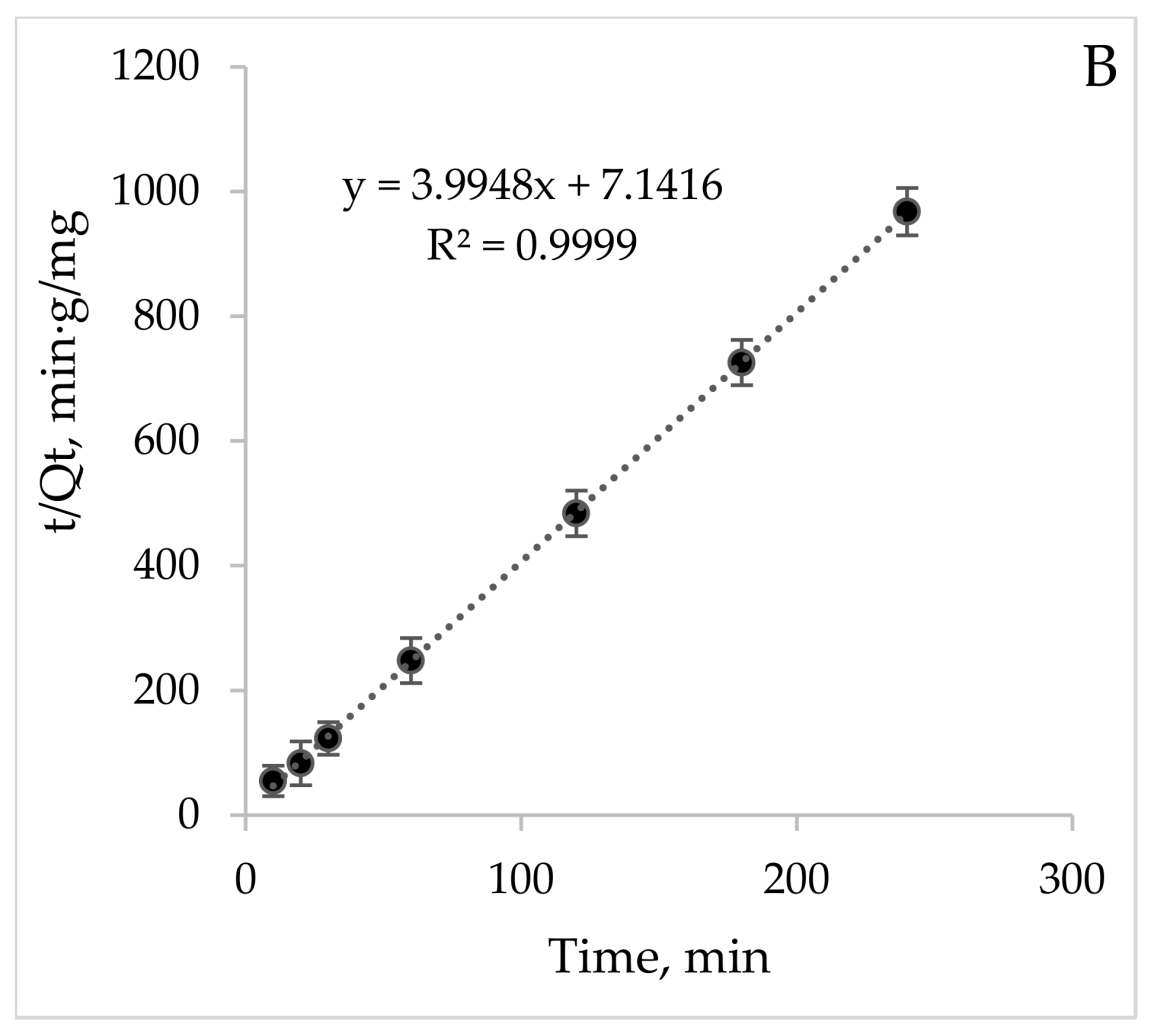
Adsorption kinetics of phenol and lead on the biochar-coated cocoon-based adsorbent: pseudo-second-order kinetics for phenol (**A**) and for lead (**B**). (error bars represent standard deviations of triplicate measurements).

To further characterize the adsorption process, the experimental kinetic data were fitted using the pseudo-second-order model (Figure 7). The calculated kinetic parameters and coefficients of determination (R^2^) are presented in Table 3. The fitted model was used to evaluate the agreement between the experimental data and the predicted adsorption behavior.

**Figure 8:**
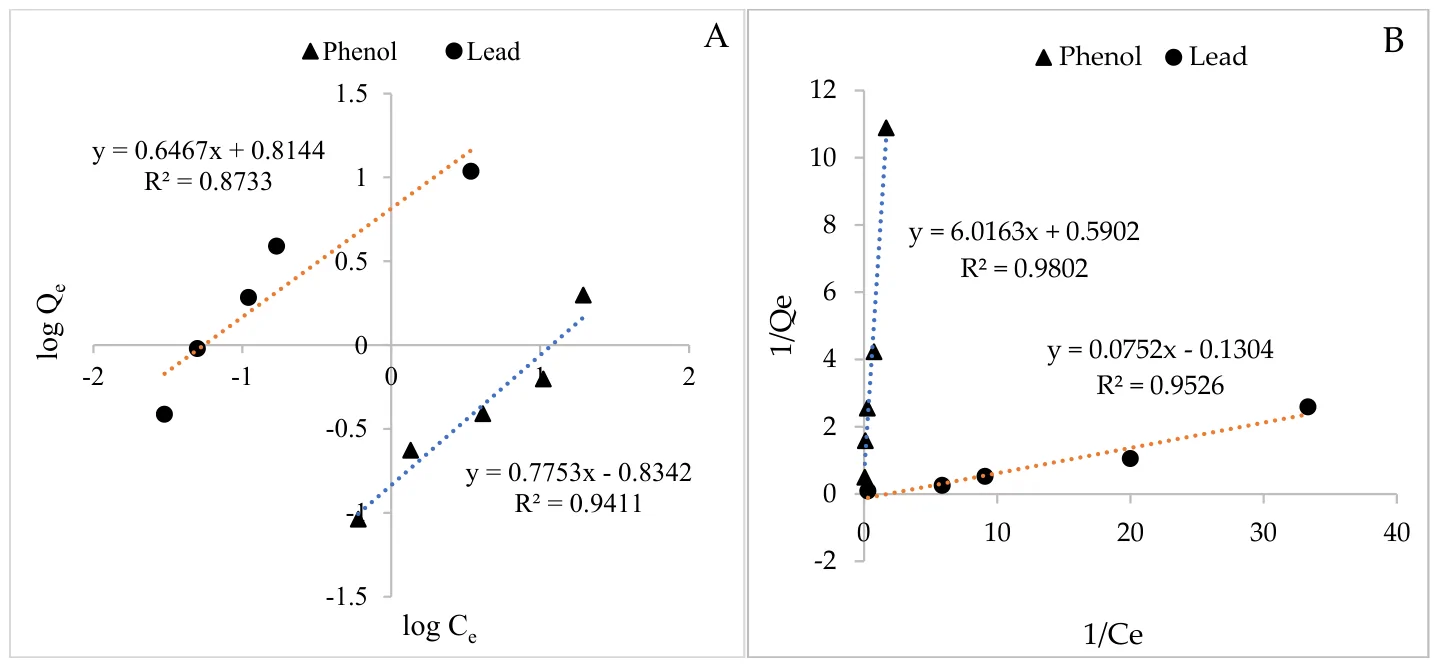
Adsorption isotherms of phenol and lead on the biochar-coated cocoon-based adsorbent: (**A**) Freundlich isotherm and (**B**) Langmuir isotherm (error bars represent standard deviations of triplicate measurements).

The adsorption equilibrium and kinetics were analyzed to better understand the adsorption mechanism and efficiency of the developed material. Equilibrium isotherm models were applied to describe the adsorption capacity and surface characteristics were used to evaluate the rate of adsorption and identify the controlling mechanisms (Figure 8).

The adsorption equilibrium of Pb(II) and phenol on the silkworm cocoon-derived carbon materials was analyzed using the Langmuir and Freundlich isotherm models. The obtained parameters are presented in Table 4.

The adsorption equilibrium of Pb(II) and phenol on the biochar-coated cocoon-based adsorbent was analyzed using the Langmuir and Freundlich isotherm models. The obtained parameters are presented in Table 4. The adsorption of Pb(II) was slightly better described by the Langmuir model (R^2^ = 0.950) than by the Freundlich model (R^2^ = 0.940). However, the relatively small difference between the correlation coefficients indicates that both models provide a comparable description of the experimental equilibrium data. Therefore, the Langmuir fit should not be interpreted as definitive evidence of strictly monolayer adsorption on homogeneous sites. The calculated Langmuir parameters were Q_m_ = 7.669 mg/g and K_L_ = 0.645 L/mg, describing the adsorption capacity and affinity of the composite surface toward Pb(II) ions. The favourable adsorption behaviour is consistent with the structural characterization of the developed composite. BET analysis demonstrated a well-developed microporous structure with a specific surface area of 422.29 m^2^ g^−1^, while SEM observations confirmed that the biochar coating uniformly covered the silkworm cocoon fibers, producing a rough surface rich in accessible adsorption sites. The Freundlich parameters (K_F_ = 6.516 and n = 1.546) also indicate favourable adsorption, suggesting that surface heterogeneity may contribute to the adsorption process. The adsorption mechanism is likely governed by surface complexation and electrostatic interactions between Pb(II) ions and oxygen-containing functional groups (-OH and -COOH) present in the biochar and alginate matrix. The FTIR analysis confirmed the presence of oxygen-containing functional groups in the activated biochar, as evidenced by the bands attributed to C-O stretching vibrations at approximately 1159 and 1042 cm^−1^. These functional groups may provide active sites for interactions with Pb(II) ions and thus contribute to their adsorption. Similarly, phenol adsorption was best described by the Langmuir model (R^2^ = 0.980), whereas the Freundlich model showed a lower correlation coefficient (R^2^ = 0.870). The calculated Langmuir parameters were Q_m_ = 1.694 mg/g and K_L_ = 1.903 L/mg. The substantially higher R^2^ value for the Langmuir model indicates that the Langmuir equation provides a more accurate empirical description of the equilibrium data than the Freundlich model. The Freundlich parameters (K_F_ = 0.146 (mg/g)(L/mg)^1/n^ and n = 1.290) also indicate favourable adsorption; however, the lower goodness of fit suggests that the Freundlich model provides a less accurate description of the experimental equilibrium data. The FTIR spectrum of the activated biochar exhibited a band at approximately 1556 cm^−1^, assigned to aromatic C=C stretching, indicating the presence of condensed aromatic carbon structures. These aromatic domains may facilitate π–π interactions with the aromatic ring of phenol. In addition, the C-O bands observed at approximately 1159 and 1042 cm^−1^ indicate the presence of residual oxygen-containing functional groups that may participate in hydrogen bonding with phenol [32].

### 3.4. Dynamic Adsorption Experiments and Regeneration of Biochar-Coated Cocoon-Based Adsorbents

Dynamic adsorption experiments were conducted to evaluate the performance of the biochar-coated cocoon-based adsorbent under flow conditions. It should be noted that the column experiments were conducted at a model concentration of 1 mg/L. Therefore, the obtained results primarily reflect the adsorbent performance under controlled experimental conditions and should be interpreted with caution when extrapolating to waters with substantially different contaminant concentrations.

**Figure 9:**
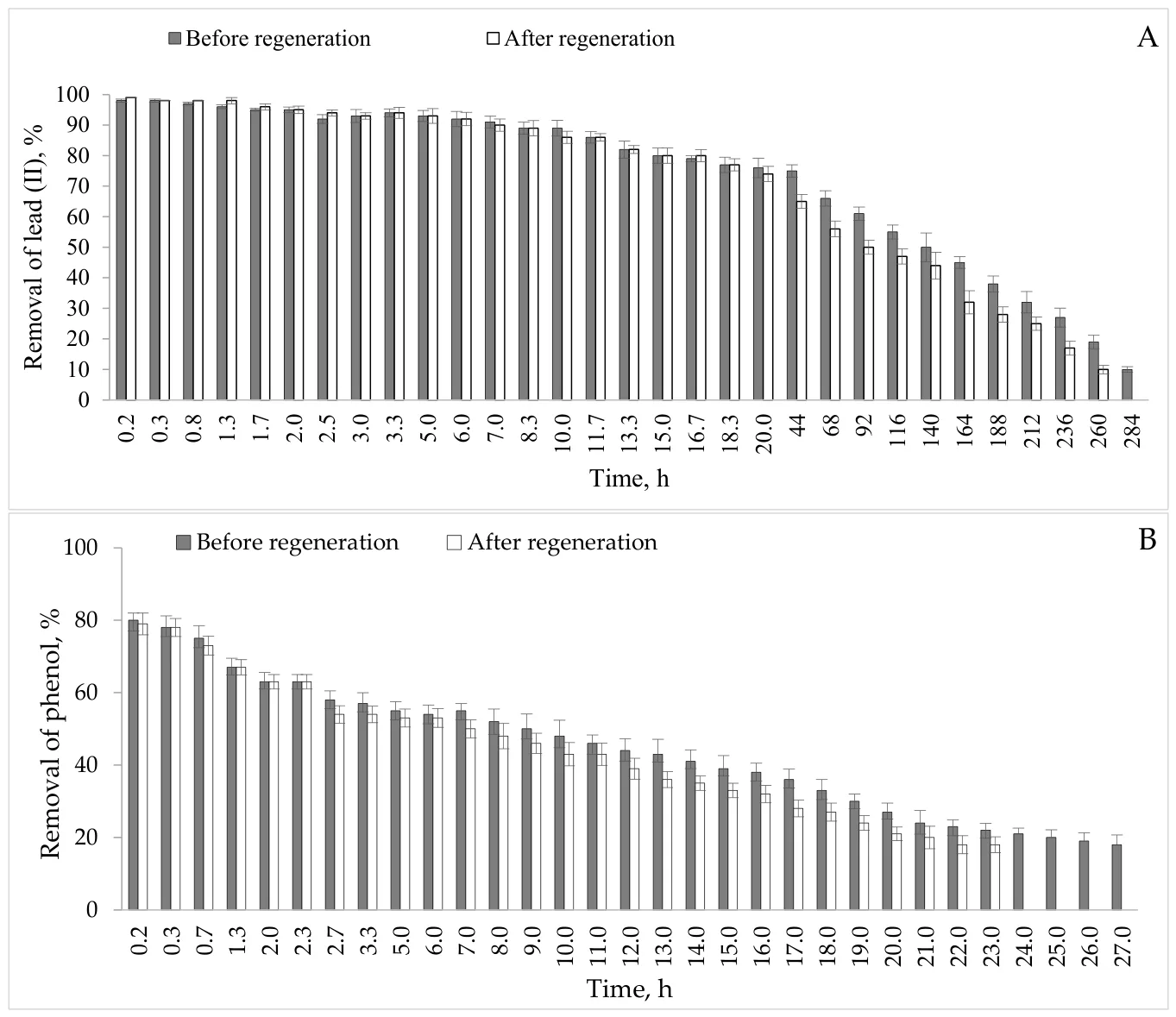
Removal efficiency of lead (II) (**A**) and phenol (**B**) in dynamic adsorption experiments for columns before and after regeneration.

A single regeneration cycle was performed to evaluate the recovery of adsorption performance. After regeneration with 0.01 M EDTA, the column showed a high removal efficiency for Pb(II), although its performance was slightly lower than that of the fresh adsorbent. In contrast, regeneration with 0.1 M NaOH only partially restored the adsorption performance for phenol. These results demonstrate partial recovery of adsorption performance after one regeneration cycle; however, further experiments involving multiple consecutive adsorption–desorption cycles are necessary to establish the long-term reusability and stability of the material. Figure 9 presents removal efficiency of lead and phenol during adsorption under dynamic conditions. In the case of Pb(II), a high removal efficiency (98%) was observed in the initial phase of column operation (0–2 h), with residual concentrations below 0.06 mg/L increasing to 0.26 mg/L after 20 h and 0.5 mg/L after 116 h, corresponding to a decrease in removal efficiency to approximately 55%. This behavior indicates a high dynamic adsorption capacity and is consistent with strong interactions between Pb(II) ions and the adsorbent surface. The FTIR analysis confirmed the presence of oxygen-containing functional groups, with bands at approximately 1159 and 1042 cm^−1^ attributed to C-O stretching vibrations. These functionalities may provide active sites for Pb(II) binding through surface complexation and electrostatic interactions, contributing to the high removal efficiency observed under dynamic conditions. After regeneration with 0.01 M EDTA, the column recovered a high adsorption efficiency, although slightly lower than that of the fresh adsorbent. This result indicates good regeneration efficiency and suggests that the developed material retains a substantial portion of its adsorption performance after regeneration. The initial removal efficiency of phenol was 80% and dropped to 63% in the first 2 h and dropped to 55% after 7 h, indicating rapid saturation of adsorption sites This difference may be related to the different nature of the interactions involved in the adsorption of Pb(II) and phenol, including surface complexation and electrostatic interactions for Pb(II), and π–π interactions and hydrogen bonding for phenol. The FTIR spectrum of the activated biochar showed a band at approximately 1556 cm^−1^, attributed to aromatic C=C stretching, indicating the presence of condensed aromatic structures that may facilitate π–π interactions with phenol. Regeneration with NaOH only partially restored adsorption capacity, confirming the limited reusability to remove phenol. Similar trends have been reported for biochar-based adsorbents, which may exhibit higher dynamic adsorption performance toward heavy metals than toward certain organic pollutants. This difference can be related to the specific interactions between metal ions and oxygen-containing surface functionalities, including electrostatic attraction and surface complexation, whereas organic pollutants such as phenol may be predominantly adsorbed through π–π interactions and hydrogen bonding. Overall, the results obtained indicate that the developed biochar-coated cocoon-based adsorbent is a promising adsorbent for the removal of Pb(II) under dynamic conditions, while its performance relative to phenol is relatively lower, but still significant.

**Figure 10:**
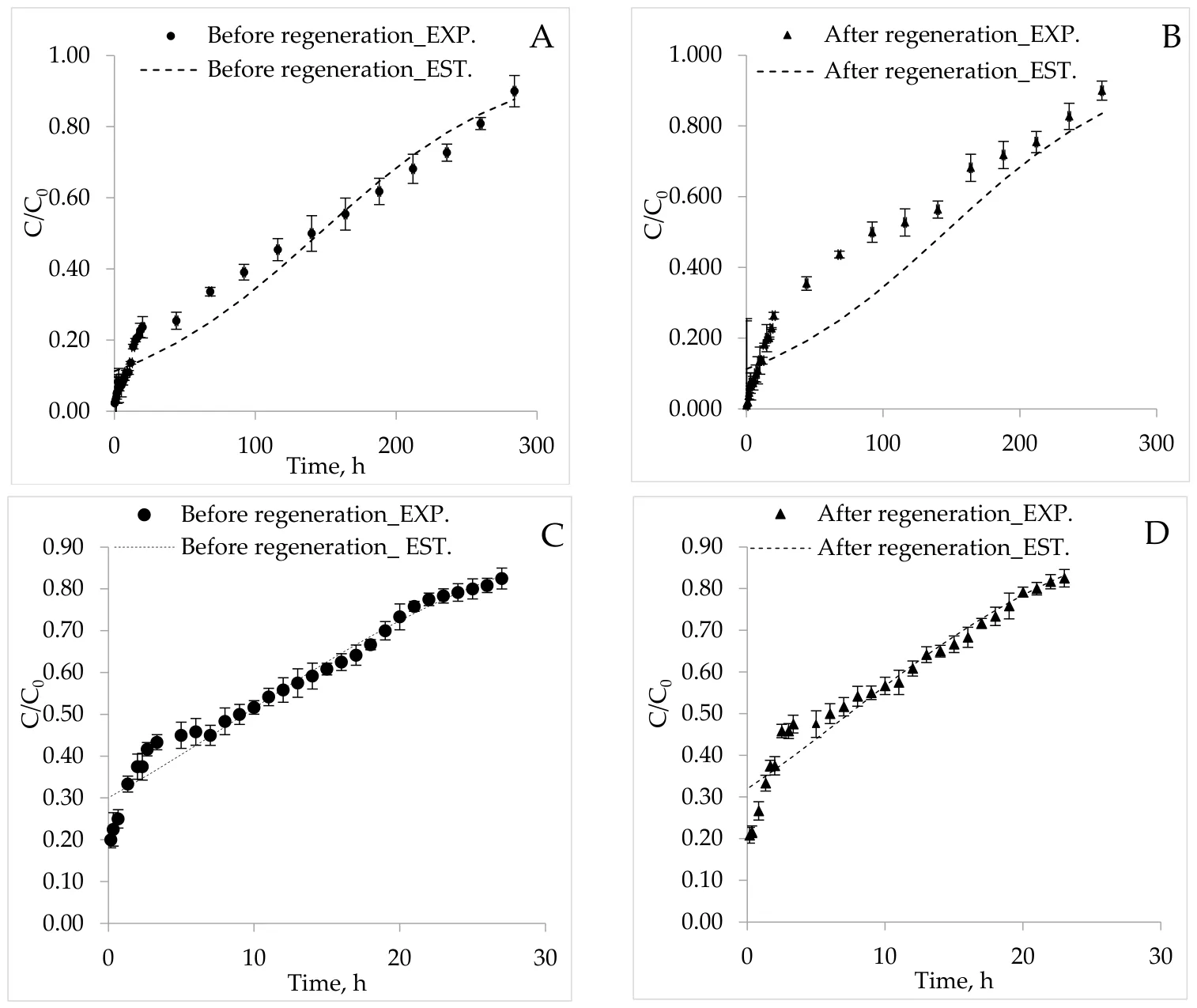
Breakthrough curves (C/C_0_) of lead (II) (**A**,**B**) and phenol (**C**,**D**) during column adsorption before and after regeneration.

Results obtained from the column experiments are also presented as breakthrough curves in Figure 10. The experimental data were described using the Thomas model, which assumes that the adsorption process follows Langmuir kinetics while neglecting intraparticle mass transfer and diffusion resistances [33]. In the initial stage of the experiments, the breakthrough curves were skewed and steep, suggesting heterogeneity of the adsorption bed. The 10% breakthrough point for lead was reached after 7 h of column operation for both fresh and regenerated adsorbents. For phenol, this threshold could not be determined because of the relatively high phenol concentration measured at the initial stage of column operation. Using the 50% breakthrough point for comparison revealed a clear advantage of lead adsorption: the breakthrough point was reached after 140 h and 92 h for the fresh and regenerated adsorbents, respectively. In contrast, for phenol, the 50% breakthrough point occurred much earlier, after 9 h and 6 h for the fresh and regenerated adsorbents, respectively. The exhaustion time was much longer for lead (284 h) than for phenol (27 h), however it decreased by a few hours after regeneration, to 260 h for lead and 23 h for phenol.

The parameters of the Thomas model are presented in Table 5. Based on the R^2^ values, it is evident that the Thomas model described phenol adsorption better than lead adsorption. This indicates that phenol adsorption followed the assumptions of the Thomas model, whereas lead (removal may have involved more complex mechanisms, such as ion exchange and electrostatic interactions [34,35]).

A comparison of the q_Th_ values, representing the maximum adsorption capacity, clearly shows that the adsorbent exhibited a much higher capacity for Pb(II) removal than for phenol adsorption. For both lead and phenol, the maximum adsorption capacity decreased by approximately 20% after regeneration. This behavior is typical, as regeneration usually allows only partial recovery of adsorption sites [36].

The higher K_Th_ values obtained for phenol indicate a faster breakthrough and more rapid saturation of the adsorption bed under dynamic conditions. This should not be directly compared with the pseudo-second-order kinetic constants obtained from batch experiments, where Pb(II) exhibited a significantly higher adsorption rate (K_2_ = 2.234 g/(mg·min)) than phenol (K_2_ = 0.249 g/(mg·min)). The differences arise because the Thomas model describes the overall column performance, including mass transfer effects, whereas the pseudo-second-order model reflects the intrinsic adsorption kinetics under equilibrium batch conditions. Furthermore, after regeneration, the adsorption process was slightly faster for both adsorbates. This phenomenon may be attributed to change in the adsorbent structure and active sites resulting in easier and faster access to adsorption sites [37]. Deng et al. reported that the rate of adsorption was faster after adsorbent regeneration [38].

## 4. Comparison with Previously Reported Biomass-Derived Adsorbents

To evaluate the performance of the developed biochar-coated silkworm cocoon composite, its adsorption properties were compared with selected biomass-derived adsorbents reported for the removal of inorganic and organic contaminants from aqueous solutions. The comparison considers adsorption capacity, textural properties, adsorbent architecture, and applicability under dynamic operating conditions, where relevant data are available.

Biomass-derived biochars prepared from agricultural and forestry residues have attracted considerable attention because of their renewability, availability, and potential environmental benefits [39,40]. Various biomass precursors, including rice husks, pine sawdust, and sewage sludge, have been used to produce adsorbents for removing heavy metals and organic contaminants from water [41,42]. Their performance depends on the precursor, activation method, and surface modification. For example, iron-modified rice husk biochar achieved removal efficiencies of 99.56% for Pb(II), 99.98% for Cd(II), and 99.96% for Hg(II) [41]. Magnetic chitosan/sewage sludge biochar exhibited a Cu(II) adsorption capacity of 55.16 mg/g [43], whereas APTES-functionalized magnetic biochar achieved adsorption capacities of 64.92 mg/g for Pb(II) and 48.86 mg/g for Cr(VI) [44]. These examples demonstrate that chemical modification can substantially enhance the adsorption performance of biomass-derived materials.

The textural properties of biochars are also strongly affected by the activation method. KOH activation is widely used to develop porous structures and increase the specific surface area of carbonaceous materials [45]. In the present study, the activated biochar-coated cocoon composite exhibited a specific surface area of 422.29 m^2^/g, lower than that reported for some highly activated biochars, including the reed-derived material listed in Table 5 [45]. However, adsorption performance cannot be assessed solely on the basis of BET surface area, because surface functionality, pore accessibility, morphology, and mass-transfer characteristics also influence pollutant uptake [39,40]. A quantitative comparison of selected biomass-derived adsorbents is presented in Table 6. The reported adsorption capacities vary considerably with the adsorbent precursor, modification method, pollutant, and experimental conditions; therefore, the (Qm) values should not be interpreted as a direct ranking of overall adsorbent performance.

Despite the high adsorption capacities reported for many modified biochars, most studies have focused on batch adsorption experiments [41,42,43,44]. Batch tests provide useful information on adsorption equilibrium and kinetics but do not fully represent the hydraulic conditions encountered in practical water treatment systems. Continuous-flow column studies remain comparatively less common, although they provide information on breakthrough behaviour and performance under dynamic operating conditions [46,47,48]. Consequently, evaluating adsorbents in column systems is important for assessing their potential applicability beyond laboratory batch experiments.

Another practical limitation of biomass-derived adsorbents is their frequent use in powdered form. Although powdered biochars can provide high equilibrium adsorption capacities because of their small particle size and large exposed surface area [49], their recovery from treated water may require additional separation steps, such as filtration or centrifugation, and particle loss may occur during operation [50]. In fixed-bed systems, powdered materials may also contribute to bed clogging, hydraulic instability, or increased pressure drop. These limitations have encouraged the development of immobilized adsorbents, including composite beads, films, hydrogels, magnetic composites, and supported biochars [51,52,53,54].

Immobilization can improve material handling and facilitate separation, although its effect on adsorption capacity depends on the composition and structure of the resulting composite. In some systems, immobilization may introduce additional adsorption sites or improve pore accessibility. For example, alginate/polyaniline composite beads exhibited an increased methylene blue adsorption capacity compared with powdered biochar [55]. Other supported biochar composites have also shown substantial adsorption capacities for organic pollutants and phosphate [53,54]. These studies indicate that structural design can be an important consideration alongside chemical surface modification when developing adsorbents for practical applications.

Silkworm cocoons and their major proteins, fibroin and sericin, have been extensively investigated as biomaterials for wound healing, tissue engineering, regenerative medicine, and drug delivery because of their biocompatibility and mechanical properties [56,57,58,59]. In contrast, their application in water treatment remains comparatively limited. In our previous study, silkworm cocoon-derived biochar was investigated for contaminant removal from rainwater [60]. The present study extends this work by immobilizing activated biochar on the natural cocoon framework. The study also examines breakthrough behaviour and performance following one regeneration cycle. Thus, the novelty of the present work lies in the integration of the biochar precursor and structural support derived from the same biomass and in the evaluation of the resulting composite under dynamic conditions, rather than in the use of silkworm cocoon-derived biochar alone. From an environmental perspective, the management of the spent adsorbent must also be considered. Following Pb(II) adsorption, the material may contain lead at levels requiring controlled handling and assessment. It should not be disposed of without appropriate evaluation, and its management must comply with applicable waste reg-ulations. Future studies should investigate lead recovery and the safe management or regeneration of the spent material. This should be assessed in future work to determine whether the composite affects treated-water quality.

Although the composite does not exhibit the highest adsorption capacities among the materials reported in the literature, its architecture and evaluation under contin-uous-flow conditions provide a different basis for assessing its potential in water treatment.

## 5. Conclusions

In this study, a novel biochar-coated silkworm cocoon composite was successfully developed and evaluated as an adsorbent for the removal of inorganic and organic pollutants from aqueous solutions. The main conclusions are as follows:

1. KOH activation significantly enhanced the textural properties of the biochar, increasing the specific surface area from 1.56 to 1178 m^2^/g and producing a predominantly microporous structure.
2. SEM, FTIR, BET, and TGA analyses confirmed the preservation of the cocoon morphology after coating, the presence of oxygen-containing functional groups, and the satisfactory thermal stability of the developed composite.
3. Adsorption studies demonstrated that the developed adsorbent effectively removed both Pb (II) ions and phenol from aqueous solutions, with considerably higher adsorption capacity and removal efficiency for Pb(II), particularly under dynamic flow conditions.
4. Equilibrium studies showed that the Langmuir model provided the best fit for both Pb(II) and phenol adsorption. However, the relatively close Langmuir and Freundlich R^2^ values for Pb(II) indicate that both models provide a comparable description of the equilibrium data, and therefore the Langmuir fit should not be considered definitive evidence of strictly monolayer adsorption.
5. A single regeneration cycle resulted in partial recovery of adsorption performance, particularly for Pb(II). Further consecutive adsorption–desorption cycles are required to evaluate the long-term reusability and stability of the composite.
6. Column experiments demonstrated substantially higher adsorption capacity, longer breakthrough time, and longer exhaustion time for Pb(II) than for phenol. Regeneration reduced the adsorption capacity by approximately 20% for both adsorbates while maintaining satisfactory column performance.
7. Compared with conventional powdered adsorbents, the developed biochar-coated silkworm cocoon composite combines high adsorption performance with improved handling, easier separation from treated water, reduced material loss, and suitability for continuous-flow systems, making it a promising, sustainable, and practical adsorbent for water treatment applications.

## Figures and Tables

**Table 1:** True density values of carbon materials derived from silkworm cocoon at different stages of processing.

Sample	True Density (g/cm^3^)
Carbonized cocoon	1.681 ± 0.004
Biochar	1.938 ± 0.008
Biochar-coated cocoon	1.657 ± 0.005

**Table 2:** Textural properties of silkworm cocoon-derived carbon materials.

Sample	S_BET_ (m^2^/g)	V_p_ (cm^3^/g)	D_p_ (nm)	The Nature of the Pores
Carbonized cocoon	1.56	0.0026	3.78	poorly developed, mesopores
Biochar	1178	0.539	1.93	microporous
Biochar-coated cocoon	422.29	0.1723	1.43	microporous

**Table 3:** Pseudo-second-order kinetic parameters for Pb(II) and phenol.

Parameter	Pseudo-Second-Order Equation Parameters		
	K_2_ (g/(mg·min))	Q_e_ (mg/g)	R^2^
Lead	2.234	0.25	0.999
Phenol	0.249	0.0794	0.968

**Table 4:** The Langmuir and Freundlich parameters for adsorption equilibrium of lead and phenol on the biochar-coated cocoon-based adsorbent.

Parameter	Langmuir			Freundlich		
	Q_m_ (mg/g)	K_L_ (L/mg)	R^2^ (-)	K_F_ ((mg/g) L/mg)^n^)	*n* (-)	R^2^ (-)
Lead	7.669	0.645	0.950	6.516	1.546	0.940
Phenol	1.694	1.903	0.980	0.146	1.290	0.870

**Table 5:** Parameters of Thomas model for adsorption equilibrium of Pb(II) and phenol in column experiments.

	Thomas Model		
Parameter	K_Th_ (L/h*mg)	q_Th_ (mg/g)	R^2^ (-)
Pb(II) (before regeneration)	0.0129	9.99	0.8032
Pb(II) (after regeneration)	0.0156	8.12	0.8197
Phenol (before regeneration)	0.0753	0.69	0.9237
Phenol (after regeneration)	0.0852	0.55	0.9061

**Table 6:** Quantitative comparison of selected biomass-derived adsorbents for the removal of inorganic and organic pollutants from aqueous solutions.

Biomass Precursor and Adsorbent Modification	Activation Method	SBET (m^2^/g)	Q_m_ for Pb(II) (mg/g)	Q_m_ for Phenol or Other Organic Pollutants (mg/g)	Experimental Conditions	Ref.
*C. odorata* roots; magnetic biochar functionalized with APTES and Fe_3_O_4_	Pyrolysis and magnetic/chemical modification	35.66	64.92	—	Pb(II): C_0_ = 10–100 mg/L; adsorbent dosage = 1 g/L; pH varied; 20 °C; 60 min	[44]
Reed-derived biochar (650-K)	KOH activation at 650 °C	965.31	—	173.61 (tetracycline)	Tetracycline: C_0_ = 60–300 mg/L; pH 3–9; 15 mg adsorbent; temperature varied from 15 to 40 °C	[45]
Date seed-derived biochar (DSB550-3)	Pyrolysis at 550 °C for 3 h	Not determined by BET	74.59 *	—	Pb(II): C_0_ = 0.3–4.0 mM; pH = 6; dosage = 10 g/L; 23 ± 2 °C; 24 h	[46]
Biochar-coated silkworm cocoon composite	Pyrolysis and KOH activation 800 °C; immobilization in sodium alginate matrix	422.29	7.669	1.694 (phenol)	Batch adsorption: room temperature; dosage varied from 1 to 6 g/L for Pb(II) and 3 to 14 g/L for phenol. Column tests: C_0_ = 1 mg/L for both pollutants	Present study

* The adsorption capacity of date seed biochar was converted from 0.360 mmol/g, as reported in the original publication, to 74.59 mg/g using the molar mass of Pb.

## Data Availability

The original contributions presented in this study are included in the article. Further inquiries can be directed to the corresponding author.

## Supplementary Materials

The following supporting information can be downloaded at: https://www.mdpi.com/article/10.3390/ma19214512/s1, Figure S1. Nitrogen adsorption-desorption isotherm of the biochar-coated cocoon-based adsorbent. Red circles represent the adsorption branch, and blue circles represent the desorption branch.
