# Supplementary material for: Innovative Biochar-Coated Cocoon-Based Adsorbent for the Removal of Organic and Inorganic Pollutants from Water
Source: Materials (Basel). 2026 Oct 27;19(21):4512. doi: 10.3390/ma19214512 (PMC13763729; doi:10.3390/ma19214512)
Supplement: Supplementary file 1 [file materials-19-04512-s001.zip › materials-4625367-Supplementary.pdf]

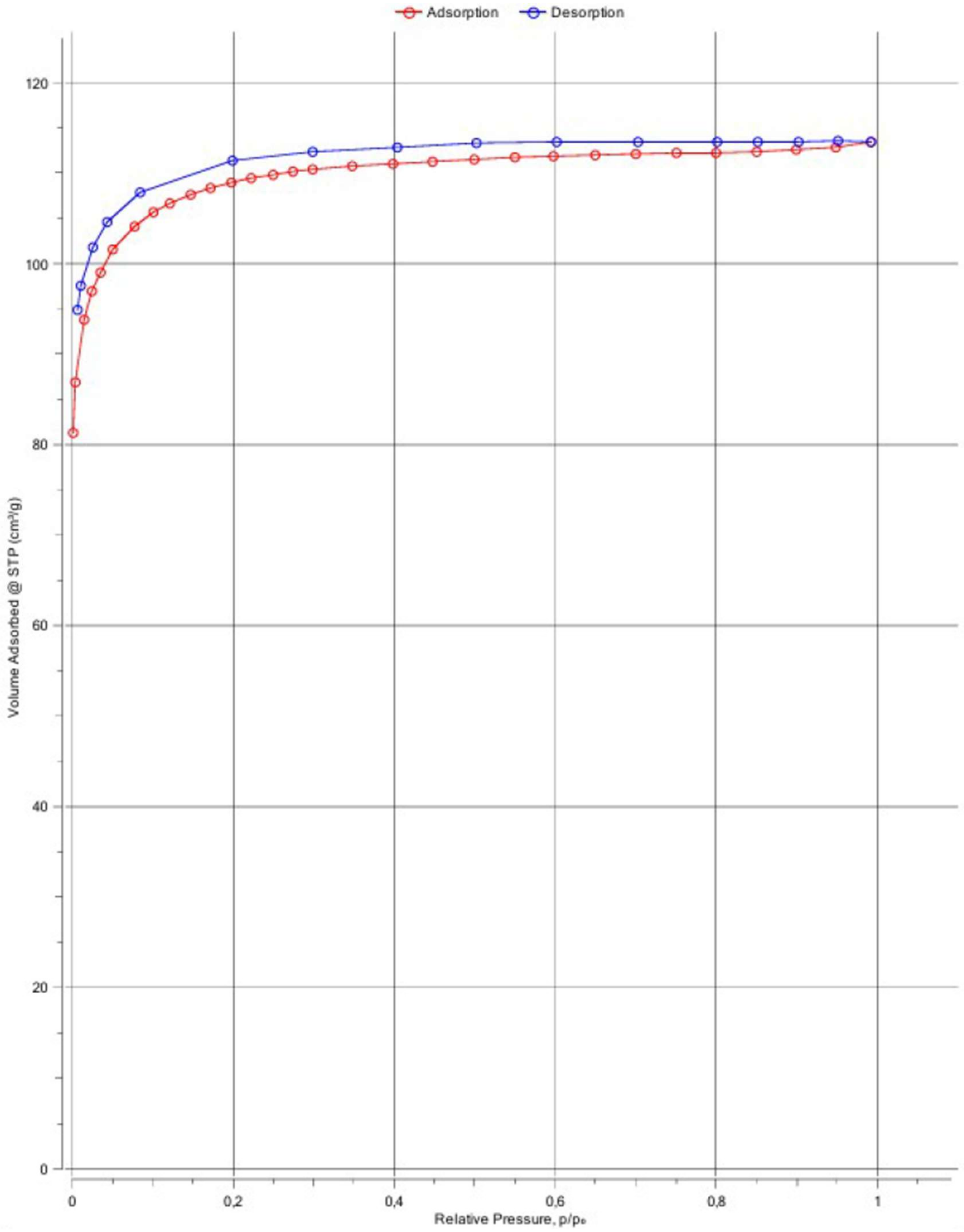

Adsorption
Desorption
Volume Adsorbed @ STP (cm³/g)
120
100
80
60
40
20
0
0
0,2
0,4
0,6
0,8
1
Relative Pressure, p/p₀
